# Natural Light Exposure, Sleep and Depression among Day Workers and Shiftworkers at Arctic and Equatorial Latitudes

**DOI:** 10.1371/journal.pone.0122078

**Published:** 2015-04-15

**Authors:** Elaine Cristina Marqueze, Suleima Vasconcelos, Johanna Garefelt, Debra J. Skene, Claudia Roberta Moreno, Arne Lowden

**Affiliations:** 1 Department of Environmental Health, School of Public Health—University of Sao Paulo, USP, Sao Paulo, Sao Paulo, Brazil; 2 Stress Research Institute, Stockholm University, Stockholm, Sweden; 3 Epidemiology, Public Health Graduate Program—Catholic University of Santos, UNISANTOS, Santos, Sao Paulo, Brazil; 4 Faculty of Health and Medical Sciences, University of Surrey, Guildford, Surrey, United Kingdom; 5 Science’s Health Department, Federal University of Acre, UFAC, Rio Branco, Acre, Brasil; University of Alabama at Birmingham, UNITED STATES

## Abstract

**Objectives:**

This study aimed to investigate the relationship between individual natural light exposure, sleep need, and depression at two latitudes, one extreme with a few hours of light per day during winter, and the other with equal hours of light and darkness throughout the year.

**Methods:**

This cross-sectional study included a sample of Brazilian workers (Equatorial, n = 488 workers) and a Swedish sample (Arctic, n = 1,273).

**Results:**

The reported mean total natural light exposure per 4-week cycle differed significantly between the Equatorial and Arctic regions. However, shiftworkers from both sites reported similar hours of natural light exposure. Short light exposure was a predictor for insufficient sleep.

**Conclusion:**

Reduced exposure to natural light appears to increase the perception of obtaining insufficient sleep. Arctic workers were more prone to develop depression than Equatorial workers.

## Introduction

Light is the most important time cue for maintaining the 24 h period of circadian rhythms in humans [1]. In the Arctic and Antarctic Circles, deprivation of natural light occurs in winter, and some studies suggest that these suboptimal light conditions are deleterious to health, sleep and mood [2, 3]. In the case of northern latitudes, for instance, lack of natural light during winter exerts a strong influence on sleep problems and depressive illness [4–9]. An adequate light exposure pattern may help to prevent or even reverse health problems associated with circadian disruption [5, 10–15].

Cardinali [16] and Benedetti and Terman [17] have pointed out that the prevalence of mood disorders increases with distance from the Equator, and that it therefore would be related to latitude. However, other studies consider latitude to have a small or insignificant impact on sleep complaints and depression [18–20]. Reasons for the mixed results may be that depressive mood might be more reactive to the seasonal variation of light exposure than sleep and rest activity patterns [21]. Another explanation is that the relationship between sleep and mood might be bi-directional, i.e. that changes in sleep during winter precede changes in mood, but also that mood changes affect certain sleep characteristics, especially sleep quality [10]. Mersch et al. [22] emphasized that other factors like climate, genetic vulnerability and social-cultural context may play a more important role than light on the prevalence of depression and seasonal affective disorder (SAD). However, there might also be an interaction between vulnerability and exposure, e.g. individuals who are more sensitive to variations in natural bright light might report more complaints of seasonal mood variations [23]. Finally, it has recently been suggested that people living at extreme latitudes may more openly express a vulnerability to seasonal change, whereas such vulnerability is latent in tropical areas [24].

In this study, we have compared two different populations of workers, one living beneath the Equator in northern Brazil and another within the Arctic Circle in northern Sweden. Our hypothesis was that at an extreme latitude (Swedish workers) lack of natural light exposure affects sleep quality and may lead to depressive symptoms. The Swedish participants live and work at an extreme latitude with a few hours of light per 24 hours during the winter time (when the data were collected), whereas the Brazilian participants live in an ideal environment in terms of natural light availability, considering there are 12 hours of darkness and 12 hours of natural light throughout the year (12:12 light-dark cycle). Thus, these workers were considered as the reference group in the analyses. The aim of the study was to investigate the relationship between individual natural light exposure and sleep complaints and depression, controlling for work conditions and other health problems, at two latitudes.

## Materials and Methods

### Participants

This cross-sectional study included a sample of Brazilian workers in the rubber industry (Equator, n = 488 workers, response rate 89%) and a Swedish sample (Arctic, n = 1,273, response rate 68%) at a mining company. Although there are many ways to calculate response rates [25], in this study we adopted the more commonly used calculation, which is to count the number of responses out of the eligible respondents (488 out of 548 in the Equatorial sample and 1273 out of 1872 in the Arctic sample). Eligible respondents in the Arctic sample encompass all workers in the mining company, and all registered rubber tappers at the rubber’s cooperative plus all workers from the factory in the Equatorial sample. A web survey was used in the Arctic. The response rate of 68% has been found to be acceptable for online surveys [26]. The Equatorial sample consisted of both outdoor working rubber tappers (n = 340) and indoor factory workers (n = 148). The research was conducted at opposing latitudes, close to the Equator in northern Brazil (latitude: 10°39'06"S; longitude: 68°30'16"W) and above the Arctic Circle in northern Sweden (latitude 67°51'20"N and longitude 20°13'30"E).

The sample formed eight groups based on work hours and latitude. The largest group (n = 719) consisted of Arctic day workers. The second group were Arctic 2-shift workers (morning/afternoon, n = 331) and the third group were working in 3-shifts (morning/afternoon/night, n = 223). The Equatorial sample of early morning workers (work starting before 06:00 h, n = 238) made up the fourth group, the fifth group included Equatorial late morning workers (work starting after 06:00 h, n = 102) and the sixth group consisted of Equatorial day workers (n = 46). The seventh group included Equatorial 2-shift workers (n = 10) and the eighth group included Equatorial 3-shift workers (n = 92).

Data collection was performed between September and November 2011 for the Equatorial sample, in a 12:12 light-dark cycle. Data from the Equatorial sample were collected using questionnaires. Researchers read aloud the questionnaires for illiterate participants.

In the Arctic, data were collected using an online questionnaire given in January 2013. The Arctic workers were instructed to report health status and light behavior based on the period November to January. Thus, Arctic workers were living in a light-dark cycle that varied from 7 hours of daylight to zero hours (ranging from 07:17 to 00:24 light-dark cycle). The period included 27 polar days and partly due to the inland Arctic climate the daily mean hours of sunshine never exceeded one hour within the three winter months (<1 kWh/m2 in global energy from sun radiation [27]. Similar solar radiation measures from the Amazon in Brazil hoover around 5 kWh/m2 [28].

### Variables

The study included two dependent variables, insufficient sleep and depression. Insufficient sleep was composed from a single item question, extracted from a validated sleep scale [29], in the questionnaires at both sites: Do you sleep enough? The response alternatives were “(5) yes, definitely”; “(4) yes, almost enough”; “(3) no, just a little”; “(2) no, too little”; “(1) no, definitely not”. The variable was dichotomized at the value ≥ 4 (yes) and ≤ 3 (no).

At both sites, clinical depression was reported. In the Equatorial sample a question regarding depression from the Work Ability Index (WAI) was used. In this validated index diseases are listed and the participants indicate if the diseases are diagnosed by a physician [30]. The WAI version used in this study was validated in Portuguese [31, 32]. In the Arctic sample, depressive symptoms were measured using a 6-item scale corresponding to the Hamilton Depression Subscale (HAM-D6) [33] that measured symptoms of: feeling blue; feeling no interest in things; feeling lethargy or low in energy; worrying too much about things; blaming yourself for things; and feeling everything is an effort [34]. Each item measured three-month prevalence by asking: “How much during the past three months has that problem troubled you?” and is quantified on a 5-category scale from 1 = not at all to 5 = extremely. A summated score of the above six items ranging from 6–30 was used for the analyses. The scale has been used as a valid measure of depression severity compared to other self-report depression scales [35], using a cut-off equal to 23 or more to indicate major depression.

Background variables included age (mean, SD), sex (female *vs*. male), smoking (no *vs*. yes), civil status (with partner *vs*. without partner), educational level (illiterate *vs*. primary school *vs*. high school *vs*. university), and groups of workers according to light exposure.

The Arctic workers reported how many hours per day on average they were exposed to natural light (not artificial light) within the period from November to January in connection to both work days and days-off. The mean total hours of natural light exposure was calculated according to the estimated number of work days and days-off. In the Arctic sample, since the work days varied according to a 4-week cycle, it was calculated to be 16/12 for shiftworkers (work days and days-off, respectively) and 20/8 for day workers.

The Equatorial workers reported how many hours per day on average they were exposed to natural light in connection to work days and weekends. Within the Equatorial sample the ratio was 20/8.

Natural light exposure was dichotomized into long and short exposure according to the relative median or mean hours within each group of workers.

Three independent indices related to sleep problems were obtained from the Karolinska Sleep Questionnaire [36]. The first index, sleep quality, was derived from four items: difficulties falling asleep, disturbed sleep, repeated awakenings, premature awakening. The second index, awakening problems, comprised of two items: difficulties awakening and not well rested on awakening. The third index, sleepiness, included three items: nodding off at work, nodding off during leisure-time, fighting sleep—an effort to remain awake. The response alternatives were: “always/every day (5)”; “mostly/several days per week (4)”; “sometimes/several times per month (3)”; “seldom/a few times per year (2)”; “never (1)”. The three indices were dichotomized at the value ≥ 4 (yes, problems) and < 4 (no problems).

Other independent variables included in the analyses were: neck and low back pain (no pain *vs*. neck or low back pain *vs*. neck and low back pain); work duration per week (< 50 hrs *vs*. ≥ 50 hrs); satisfaction with working time (very satisfied (1)—very unsatisfied (5)), and satisfaction with leisure-time (no *vs*. yes).

### Statistics

A chi-square test was applied to compare the Equatorial and Arctic sample proportions for sociodemographic factors (gender, civil status, education level), lifestyle (smoking), health (insufficient sleep, depression, neck and low back pain), work conditions (work hours, work duration, satisfaction with work time and leisure-time) and sleep variables (sleep quality, awakening problems and sleepiness).

To analyze the differences between means of natural light exposure, for each group of workers, we performed a one-way ANOVA with post-hoc Bonferroni correction. To compare work days and days-off within each group we conducted a repeated measures ANOVA.

A logistic regression was performed with light exposure as independent variable. We also included a linear regression analysis with the Arctic workers only, and a logistic regression with the Equatorial workers. In these separate analyses light exposure was also an independent variable.

Insufficient sleep and depression data were analyzed using a Poisson (robust variance) and logistic regression analysis yielding incidence rate ratios (IRR) and odds ratios (OR), respectively. Poisson measures were used to gain a better fit of the model due to the high prevalence of insufficient sleep [37, 38]. The predictor variables with a probability level of less than 20% were then entered into a multiple regression analysis with simultaneous adjustment for all predictor variables. In regression analyses the cut-off for natural light exposure was the relative median for each group of workers according to light exposure.

All tests were considered statistically significant when P<0.05. All data analysis was carried out using Stata, version 12.0 software package.

The study was approved by the Research Ethics Committee of the School of Public Health, University of São Paulo, Brazil (protocol # 2273) and the Regional Ethical Review Board, Stockholm, Sweden (protocol # 2012/2145-31/3) in accordance with the ethical standards laid down in the 1964 Declaration of Helsinki and its later amendments. All subjects provided written informed consent. Data available from the Dryad Digital Repository: (http://dx.doi.org/10.5061/dryad.73f69).

## Results

The Equatorial and Arctic groups had a similar mean age (37.5 ± 14.1 *vs*. 38.3 ± 11.9 yrs, P>0.05) and proportion of males (79.5% *vs*. 77.8%, P>0.05).

There were more Equatorial workers than Arctic workers without a partner, with a low education level, smoking, doing night work, and working 50 or more hours/week. In general the Equatorial group was more dissatisfied with their work hours, had worse sleep quality and reported more sleepiness (Table 1). However, they were more satisfied with their leisure time, had less neck / low back pain, less depression, less insufficient sleep and less awakening problems than the Arctic group.

**Table 1:** Sociodemographic and health data of Equatorial and Arctic workers.

**Variables**	**Categories**	**Equatorial (n = 488)**		**Arctic(n = 1,273)**		**P-value**
		**n**	**%**	**n**	**%**	**χ** ^2^
Civil Status	*With partner*	305	62.5	940	74.3	
	*Without partner*	183	37.5	325	25.7	<0.01^a^
Education level	*Illiterate*	93	19.1	0	0	
	*Primary school*	243	49.8	64	5.1	
	*High school*	127	26.0	687	54.2	
	*University*	25	5.1	516	40.7	<0.01^a^
Smoking	*No*	300	61.5	1,116	88.4	
	*Yes*	188	38.5	146	11.6	<0.01^a^
Neck / low back pain	*No pain*	284	58.2	323	25.6	
	*Neck OR low back pain*	121	24.8	395	31.3	
	*Neck & low back pain*	83	17.0	543	43.1	<0.01^a^
Depression	*No*	471	96.5	1.186	93.3	
	*Yes*	17	3.5	85	6.7	0.01^a^
Work hours	*Others*	158	32.4	1,050	82.5	
	*Night shiftwork or early morning*	330	67.6	223	17.5	<0.01^a^
Work duration / week	*< 50 h*	323	66.3	1,131	89.1	
	*≥ 50 h*	164	33.7	139	10.9	<0.01^a^
Satisfaction work time	*Very satisfied*	200	41.0	444	34.9	
	*Satisfied*	117	24.0	603	47.4	
	*Neither well nor badly*	89	18.2	129	10.1	
	*Pretty unsatisfied*	36	7.4	77	6.1	
	*Very unsatisfied*	46	9.4	20	1.6	<0.01^a^
Satisfaction leisure-time	*Yes*	286	58.6	671	52.7	
	*No*	202	41.4	602	47.3	0.03^a^
Insufficient sleep	*No*	354	72.5	809	63.6	
	*Yes*	134	27.5	463	36.4	<0.01^a^
Sleep quality (index)	*Good*	449	92.0	1,187	94.5	
	*Poor*	39	8.0	69	5.5	0.05^a^
Awakening problems (index)	*No*	460	94.3	1,124	89.4	
	*Yes*	28	5.7	133	10.6	<0.01^a^
Sleepiness (index)	*No*	449	92.0	1,237	97.8	
	*Yes*	39	8.0	28	2.2	<0.01^a^

^a^p < 0.05

The reported mean of total natural light exposure per 4-week cycle differed significantly between the Equatorial and Arctic regions (68.7 ± 44.5 h *vs*. 15.2 ± 6.9 h, P<0.01). In Table 2, natural light exposure is shown in relation to latitude and to the groups of workers. All participants from the Arctic sample (day workers; 2-shift workers; 3-shift workers) worked indoors and obtained an average amount of natural light exposure ranging from 15.7–16.4 h/4-week cycle. These levels were comparable to the Equatorial 2-shifts and 3-shifts workers (13.6 h/4-week cycle and 17.0 h/4-week cycle, respectively, P<0.05). By contrast, the Equatorial early morning, the Equatorial late morning (both groups worked outdoors) and the Equatorial day workers were, on average, exposed to more light at work days and days-off (88.3 h/4-week cycle, 88.1 h/4-week cycle and 47.6 h/4-week cycle, respectively) than the other groups (P<0.01). Furthermore, the Equatorial early morning and Equatorial late morning workers were exposed to more light at work days and days-off than the Equatorial day workers (P<0.01), the latter worked indoors. Light exposure decreased during days-off for the Equatorial non-shiftworkers and for the Arctic day workers (P<0.01, Table 2).

**Table 2:** Natural light exposure (hours/4-week cycle) of groups of workers according to light exposure.

**Natural light exposure (hours/4-week cycle)**	**Arctic day workers (indoor) n = 719**	**Arctic 2-shift workers (indoor)** ^a^ **n = 331**	**Arctic 3-shift workers (indoor)** ^b^ **n = 223**	**Equatorial early morning workers (outdoor)** ^c^ **n = 238**	**Equatorial late morning workers (outdoor)** ^d^ **n = 102**	**Equatorial day workers (indoor) n = 46**	**Equatorial 2-shift workers (indoor)** ^a^ **n = 10**	**Equatorial 3-shift workers (indoor)** ^b^ **n = 92**	**ANOVA P-value**
Median Work days & Days-off (h)	11.8	14.6	14.6	90.3	90.3	33.1	13.0	13.5	
Mean (SD) Work days (h)	16.3 (9.2)*	14.2 (7.4)	15.8 (8.1)	111.4 (48.1)*	109.7 (51.5)*	55.6 (44.9)*	13.0 (9.3)	17.6 (15.7)	<0.01
Mean (SD) Days-off (h)	10.8 (4.1)	17.4 (6.2)	17.1 (6.6)	30.5 (18.0)	29.4 (18.3)	27.7 (16.4)	15.1 (9.2)	15.6 (11.0)	<0.01
Mean (SD) Work days & Days-off (h)^e^	14.6 (7.2)	15.7 (6.1)	16.4 (6.8)	88.3 (36.2)	88.1 (38.7)	47.6 (34.4)	13.6 (8.2)	17.0 (13.5)	<0.01

^a^ Alternating morning/afternoon shifts

^b^ Alternating morning/afternoon/night shifts

^c^ Starting time before 06:00 h

^d^ Starting time after 06:00 h

^e^ Weighted averages according to the number of work days and number of days-off

* Difference mean between work days and days-off within group, P<0.01

**Fig 1:**
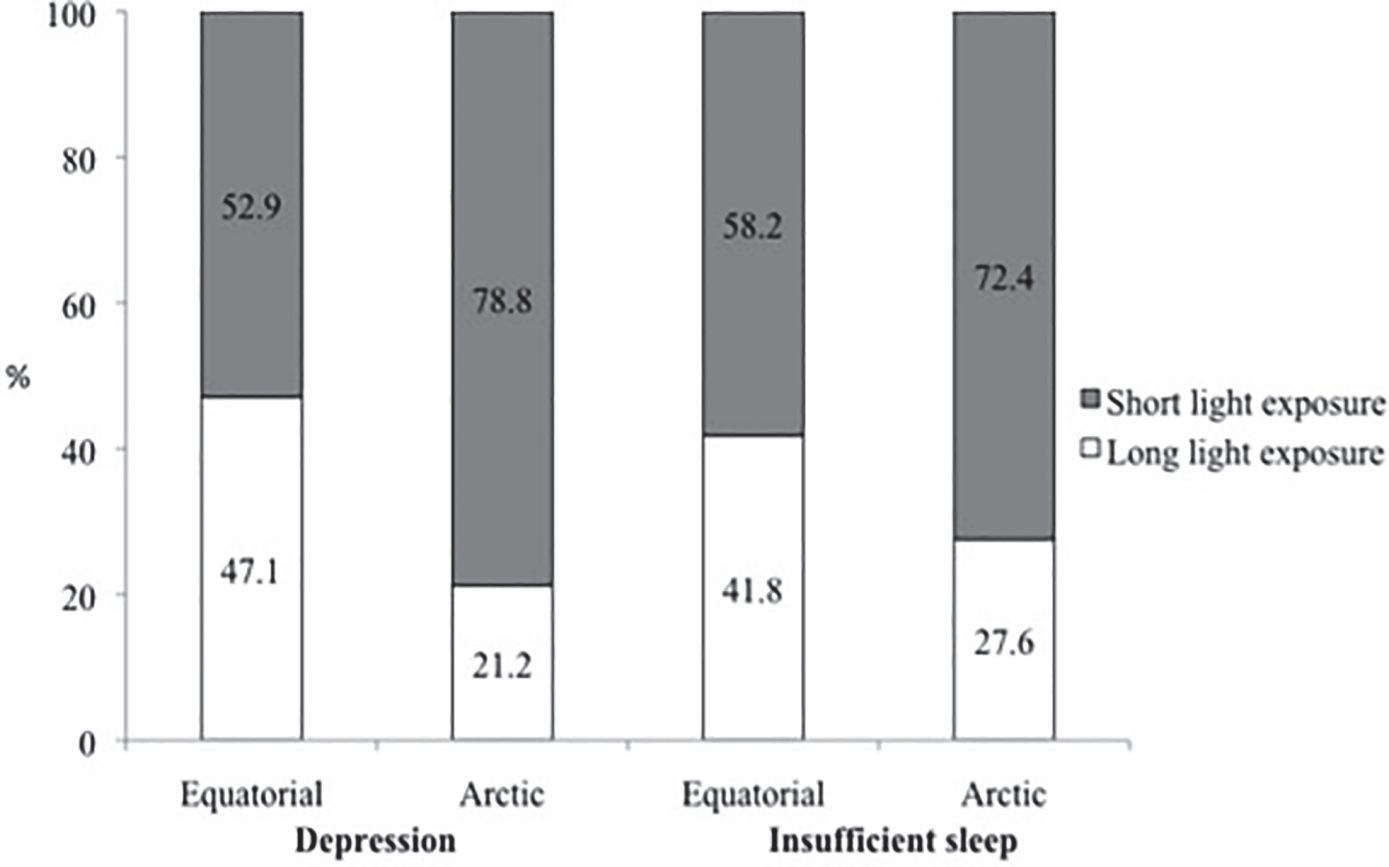
Percentage of workers reporting depression and insufficient sleep divided according to self-reported natural light exposure groups for Equatorial and Arctic groups.

A higher proportion of Arctic workers complained that they did not obtain enough sleep (36.4%) and were depressed (6.7%) compared with the Equatorial workers (27.5% and 3.5%, respectively, P<0.05). In the next step, we split the workers into short and long light exposure groups, according to the mean within each group. We found a higher percentage of depressed workers and those reporting insufficient sleep in the short light exposure groups (Fig. 1).

A linear regression analysis was conducted in order to verify the correlation between depression and natural light exposure at the northern latitude on work days only. The results demonstrated that a reduction of one hour of light exposure on work days gave an increase of 0.09 points on the depression scale (P<0.01). By contrast, the logistic regression analysis to verify an association of depression with light exposure was not significant with only the Equatorial sample.

Natural light exposure was included as a factor and latitude was used to adjust the model (among other variables—see Table 3) with both datasets (Equatorial and Arctic). In this model, short natural light exposure became a predictor for insufficient sleep, but not for depression (Table 3). All other independent variables associated with depression and insufficient sleep in the previous models came out as predictors in this model (Table 3).

**Table 3:** ORs and IRRs (with 95% CI) from crude and multiple regressions against depression and insufficient sleep, respectively (included natural light exposure).

**Variables**	**Categories**	**Depression**		**Insufficient sleep**	
		**OR (CI) Crude**	**OR (CI) Multiple** ^1^	**IRR (CI) Crude**	**IRR (CI) Multiple** ^1^
Natural light exposure in work days (median 4-week cycle)	*Long exposure*	1		1	1
	*Short exposure*	1.68 (1.03–2.75)*		1.48 (1.22–1.80)*	1.24 (1.01–1.53)*
Neck / low back pain	*No pain*	1	1	1	1
	*Neck or low back pain*	3.32 (1.74–6.36)*	2.47 (1.23–4.95)*	1.34 (1.08–1.67)*	1.20 (0.95–1.51)
	*Neck & low back pain*	4.32 (2.33–8.00)*	2.69 (1.36–5.32)*	1.79 (1.47–2.19)*	1.45 (1.16–1.81)*
Work duration / week	*< 50 h*	1		1	
	*≥ 50 h*	1.19 (0.72–1.97)		0.95 (0.76–1.17)	
Satisfaction working time	*Very satisfied*	1	1	1	1
	*Satisfied*	2.04 (1.17–3.54)*	1.44 (0.78–2.65)	1.78 (1.44–2.19)*	1.47 (1.18–1.83)*
	*Neither well nor badly*	3.50 (1.84–6.65)*	2.36 (1.15–4.84)*	2.10 (1.61–2.74)*	1.63 (1.23–2.16)*
	*Pretty unsatisfied*	4.27 (2.04–8.92)*	2.50 (1.10–5.66)*	3.24 (2.44–4.30)*	2.31 (1.71–3.11)*
	*Very unsatisfied*	3.90 (1.57–9.65)*	2.44 (0.79–7.49)	2.51 (1.72–3.66)*	2.18 (1.45–3.26)*
Satisfaction leisure-time	*Yes*	1	1	1	1
	*No*	4.17 (2.61–6.65)*	2.47 (1.47–4.14)*	1.97 (1.67–2.32)*	1.41 (1.17–1.69)*
Sleep quality (index)	*Good*	1	1	1	1
	*Poor*	5.03 (2.99–8.47)*	3.54 (1.91–6.57)*	2.21 (1.73–2.81)*	1.68 (1.30–2.17) *
Awakening problems (index)	*No*	1		1	1
	*Yes*	3.59 (2.21–5.83)*		2.18 (1.76–2.68)*	1.47 (1.17–1.85)*
Sleepiness (index)	*No*	1	1	1	
	*Yes*	4.79 (2.56–8.97)*	4.30 (1.97–9.39)*	1.77 (1.28–2.45)*	

*P<0.05

^1^Adjusted by age, sex, smoke, civil status, education level, occupation, work hours and latitude.

## Discussion

The present study identified extreme latitude and indoor work as important factors for reducing natural light exposure. The outcome measures in light exposure for the Arctic group are in agreement with previous studies that have highlighted the importance of geographical location [18–20]. Even though the Equatorial group reported more natural light exposure than the Arctic group, subgroups of shiftworkers in both regions had low natural light exposure. This is in agreement with the low light exposure levels found outside in winter and at work in connection to shiftwork [39]. In total, the natural light exposures reached similar durations of about 3.3–4.4 h per week for indoor shiftworkers at both geographical locations. The short duration of natural light exposure found among shiftworkers was partly expected, considering they worked at night, and slept during the day preventing light exposure. However, the variation in light exposure on work days and days-off between indoor shiftworkers from both latitudes and the Equatorial outdoor workers reached a five-fold difference. Our data furthermore demonstrate that during days-off the workers were not necessarily exposed to more natural light, on the contrary light exposures were found to be longer during work days than during days-off for indoor day workers and outdoor workers. Thus the notion that workers may try to compensate for reductions of light exposure during work days by increasing light exposure during days-off was not confirmed.

The rationale for using a relative dichotomization of light exposure within the groups was to clarify whether the variation in light exposure relates to outcome scores within the groups. This was possible since relatively low and high light exposure groups were found at both latitudes. We can expect that the Equatorial sample will not vary in outcome variables according to season [40] but we still do not know if subgroups, for example, indoor workers are sensitive to relatively low light exposure levels of natural daylight. Field studies to investigate the impact of extreme natural light conditions on sleep and mental health (e.g. depression) are extremely rare in the literature and currently we have little information regarding light and health [41] and what the ideal cut-off levels of light might be. Not only does natural daylight via ocular photoreceptors affect circadian regulation, the arousal system and hippocampal structures involved in mood regulation [42], but also the build up of vitamin D has been related to mental health [43] and exposures to sunlight might also affect wellbeing through the release of beta-endorphin [44]. Thus, one of the aims of the study was to provide information from an extreme light condition (Arctic environment) and, at the same time, to study the impact of natural light in a condition with equal hours of light and darkness (Equatorial environment). We therefore decided to use a dichotomization of light exposure as a procedure to split the subjects into a short and a long light exposure group within all the studied groups. This knowledge may be used to extract guidelines of natural daylight exposure strategies for promoting health. It would, for example, be of interest to test whether increased light exposure on days-off would be a valuable and healthy strategy independent of latitude.

In the current study reported problems on sleep-related items had considerable prevalence in both the Equatorial and Arctic samples but we found a significantly higher proportion of Arctic workers reporting that they did not obtain enough sleep and had depression than Equatorial workers. Prevalence levels were in agreement with earlier observations at non-extreme latitudes [6, 7, 20, 45]. Indeed, our findings also show an association of short natural light exposure with reported insufficient sleep, independent of latitude. The importance of natural light exposure for outcomes of sleep disturbances is also supported by earlier studies [3, 46, 47]. Due to a modern lifestyle and long work hours, involving shiftwork and the dependence of artificial lighting, a decline of sleep duration has been observed in the past century and population data indicate complaints of not obtaining enough sleep [48, 49]. A modern lifestyle also implies less outdoor activity. In US, according to two recent studies, the prevalence of insufficient sleep was reported to be 27% and 23%, respectively [50, 51]. It may be argued, however, that the prevalence of depression in the Equatorial group in our study (3.5%) seems low compared to previous studies [52, 53]. The World Mental Health Survey found a lifetime prevalence of depression of 14.6% in developed countries, and 11.1% in developing countries [52]. A population-based study in a small community in Brazil showed the prevalence of depression in adults to be 7.5% (95% CI 5.9–9.1) [53]. The discrepancy between this study and the current group could likely be due to the current sample being part of an active work force and not on sick leave.

In this present study an association between depression and the reduction of light exposure was only observed in the workers working in the northern latitude. Park et al. [21] pinpointed that mood, rather than activity or sleep, was likely to be more sensitive to the seasonal variation of natural light exposure. Therefore it is possible that depressive symptoms vary more in the Arctic group than in the Equatorial group according to season. In future studies it would be of interest to investigate whether Arctic workers show a reduction of depressive symptoms during summer, and perhaps reach even lower levels than groups of Equatorial workers as reported by Küller et al. [54] studying day workers across four seasons.

The measure “clinical depression” was used at both study sites. In the Equatorial sample this was obtained from the Work Ability Index, in the Arctic sample this was obtained from the Hamilton Depression Subscale. Both questionnaires (the Work Ability Index and the Hamilton Depression Subscale) are valid measures for “clinical depression” [31, 32, 35]. Furthermore, these scales are considered by several researchers as the best instruments to investigate clinical depression in a field study. Since the Equatorial sample was diagnosed by a physician and reported through the Work Ability Index while the Arctic workers answered the subscale of Hamilton Depression scale, some caution needs to be exercised when making comparisons between the study groups.

As shown in the regression analysis several factors became significant predictors for insufficient sleep. Among these were neck and low back pain, the strongest predictor being the combination of both types of pain sources. It has been established that a number of sleep dimensions are adversely related to chronic pain from the neck and lower back [55]. In the present study comparisons between geographical locations are difficult since the reports of neck and back pain from the Equatorial group might be under-estimated as the prevalence of such symptoms has been shown to be lower among urban, low income populations [56].

It was not surprising to find that the psychosocial factors of work and leisure satisfaction were significant predictors of depression and insufficient sleep. We may expect that depression and insufficient sleep are closely associated and may be mediated by demands at work. It has recently been demonstrated that increased work demands are linked to sleep problems but only weakly mediate the development of depression [34]. Thus it seems reasonable that depression and insufficient sleep can be viewed as separate entities.

Health and a feeling of wellbeing might be promoted by natural light through a mechanism involving strengthening circadian alignment to the natural dark-light cycle. Circadian alignment is affected by the timing of light exposure and the circadian strength given by natural light exposure. This view is supported by other findings indicating that the lack of natural light exposure can be deleterious to health [2, 3]. Reduced natural light exposure is not only experienced by night shiftworkers [57, 58] but also by day-workers, who have reported sleep and mood complaints at low levels of natural light exposure [46, 47]. Even though earlier studies suggested that natural light exposure could be a key factor in promoting health, for example in studies identifying latitude correlated to sleep disturbances and depression, the mechanism explaining a higher prevalence of health problems may also be influenced by other factors. Lack of natural light exposure could imply that people reduce outdoor activity, such as physical activity and social activities, increasing complaints about sleep- and emotion-related states. In the present study apart from natural light exposure, musculoskeletal problems and work hour/leisure satisfaction were also associated with sleep and depression. Considering that natural light also reduces depressive symptoms, illumination levels at workplace have to be taken into consideration. The illumination strength of the sun is far stronger when close to its zenith. Arctic workers are helped by the reflection of light in the snow during the polar day and from having less shade from vegetation, but still outdoor illumination levels differ greatly according to latitude and climate (five-fold difference as captured by measures of global solar energy, kW/m2). Thus, even though Equatorial and Arctic indoor workers received a comparable duration of light exposure, a generally lower prevalence of depression at the Equator than in the Arctic would be expected if the different characteristics of natural light exposure at the studied locations were considered. This could partly explain the lower prevalence of depression observed among the Equatorial workers compared with the Arctic workers.

There are several strategies that could improve health status at workplaces. One strategy is to provide artificial bright light. Some studies have found that bright light at work has improved performance, reduced daytime sleepiness, and improved night-time sleep quality in day-workers [58–60]. Likewise for shiftwork the beneficial effect of bright light has been demonstrated in night work, with increased levels of alertness at work [58, 60] and a reduction in the circadian misalignment associated with night work [39, 61–63]. In the present study, the Equatorial rubber tappers had higher natural light exposure than the other workers, due to their outdoor work. Accordingly one additional innovative health promoting strategy would be to increase time outdoors, by including breaks during the work hours that can be spent outdoors, for instance. This non-pharmacological intervention could likely prevent, minimize or even reverse the health problems associated with circadian disruption and sleep deprivation [5, 14, 15].

Although our findings have shown an association between short natural light exposure and insufficient sleep, it is important to highlight that there might be a number of factors associated with this outcome. In the present study, for example, workers also reported neck and low back pain problems, and poor sleep quality. This finding supports recent evidence of a bi-directional relationship between sleep and low back pain [64], suggesting that sleep improvements might reduce the perception of pain. Dissatisfaction with leisure time and sleepiness were also related to depression in the current study. It is possible that the timing and length of leisure influence mood and, consequently, may lead to symptoms of depression. A reduction of natural light therefore seems to be a consequence of the environment, work timing and behaviours that are common to groups that are considered to adhere to a modern lifestyle.

Since the study was cross-sectional we cannot determine the cause or consequence of insufficient sleep and depression. Moreover, this study does not include artificial light measurements at the workplaces or at the workers’ home, which could influence sleep and mental health. We do not have objectives measures of sleep and only one season was evaluated in Sweden. Nonetheless, we hope that these initial findings will stimulate additional future studies. The effect of light exposure varies greatly between individuals as does susceptibility for the negative health effects [23]. Seasonal variation at latitudes may enhance these individual differences. Since we did not follow individual light exposure or take into consideration the photic history of the study participants, future studies designed to evaluate these aspects are warranted. As the natural light exposure was determined subjectively, future studies should measure light exposure objectively using ambulatory light meters combined with sleep actigraphy.

In summary, short natural light exposure during work days was shown to be associated with the perception of insufficient sleep. Workers at high latitudes have a higher chance to develop depression than workers living near the Equator. Our findings suggest that the association between natural light exposure and sleep- and emotion-related issues should be seriously considered along with other psychological factors influencing sleep and mental health.
